# Detection of Extracellular Vesicles with Colocalized Surface Markers via a Capture–Release–Capture Strategy for Treatment Monitoring in Ewing Sarcoma

**DOI:** 10.1002/adhm.202505917

**Published:** 2026-02-22

**Authors:** You-Ren Ji, Yong Ju, Hui Kong, Yaya Xu, Chen Zhao, Ryan Zhang, Yue Ma, Lynn L. Zheng, Lucy R. Shi, Alex Wu, Lu-An Lin, Carina Peijia Wu, Audrey Qian, Emily Ren, Christine Zhang, Kenny Vo, Sarah Dry, Joseph G. Crompton, Noah Federman, Yazhen Zhu, Steven J. Jonas, Hsian-Rong Tseng, Shaohua Lu, Junseok Lee

**Affiliations:** 1 California Nanosystems Institute, Crump Institute for Molecular Imaging, Department of Molecular and Medical Pharmacology, David Geffen School of Medicine, University of California, Los Angeles (UCLA), Los Angeles, California, USA; 2 Department of Pathology, Zhongshan Hospital, Fudan University, Shanghai, P. R. China; 3 Department of Pathology and Laboratory Medicine, David Geffen School of Medicine at UCLA, Los Angeles, California, USA; 4 Department of Pediatrics, David Geffen School of Medicine, UCLA, Los Angeles, California, USA; 5 Division of Surgical Oncology, David Geffen School of Medicine, Los Angeles, California, USA

**Keywords:** cancer diagnosis, colocalized surface markers, Ewing sarcoma, extracellular vesicles, liquid biopsy

## Abstract

Ewing Sarcoma (ES) is a rare but aggressive malignancy of bone tissue in adolescents and young adults, where early detection of progression and real-time treatment monitoring remain unmet clinical needs. Tumor extracellular vesicles (EVs) carry surface markers and nucleic acid cargo that can serve as minimally invasive biomarkers, but single-marker EV assays often lack specificity, and colocalized-marker approaches may suffer from low sensitivity. Here, we report the ES EV Capture–Release–Capture (CaReCa) assay, a two-step enrichment strategy that combines desthiobiotin (DTB)-mediated capture/release of CD99^+^ EVs with click chemistry-mediated recapture of CD99^+^ /B7-H3^+^ EVs, introducing molecular specificity to suppress background signals. To overcome limited yield from EVs with colocalized markers, we incorporated RT-digital PCR quantification of encapsulated *ACTB* mRNA, a stable housekeeping transcript, as a sensitive proxy for EV abundance. Using only 100 μL of plasma, the ES EV CaReCa assay distinguished ES patients (*n* = 20) from healthy donors (*n* = 20) with an AUROC of 0.98. Longitudinal analysis further demonstrated that dynamic changes in the assay readouts paralleled disease progression and treatment response, consistent with PET/CT findings. Together, these results establish CaReCa as a sensitive, specific, and scalable liquid biopsy platform with translational potential for noninvasive monitoring of ES patients.

## 1 | Introduction

The promise of precision medicine relies on diagnostic approaches capable of accurately characterizing a patient’s disease state in a minimally invasive manner while capturing dynamic changes over time. Conventional diagnostic tools, such as tissue biopsies and radiographic imaging, remain the gold standard in clinical practice but have notable limitations.

Biopsies are restricted by anatomical accessibility and provide only a static snapshot of tumor evolution [1], while imaging often detects disease only after significant progression and provides limited molecular resolution [2]. These challenges have motivated the development of “liquid biopsy” solutions, which analyze tumor-derived biomarkers present in accessible body fluids such as blood, urine, or cerebrospinal fluid [3, 4]. By enabling serial assessment of biological analytes, such as circulating tumor DNA (ctDNA), circulating tumor cells (CTCs), and extracellular vesicles (EVs), liquid biopsy has emerged as a transformative tool for earlier detection, longitudinal disease tracking, and personalized therapeutic guidance.

Among these analytes, EVs have gained particular attention as versatile biomarkers for cancer detection and monitoring [5–7]. EVs are nanoscale, lipid bilayer-enclosed vesicles secreted by nearly all cell types [8, 9], with tumor cells and those within the tumor microenvironment secreting EVs in greater abundance [10, 11]. Tumor-derived EVs appear at early stages of tumorigenesis and increase throughout disease progression, with circulating levels reaching 10^8^–10^10^ EVs per mL of plasma [12, 13]. Tumor EVs faithfully reflect their cells of origin by displaying surface protein markers and carrying nucleic acids, proteins, and lipids that recapitulate the molecular state of the tumor [14]. Their nanoscale size and structural stability also allow them to persist in biofluids, with storage at −80°C preserving both vesicles and their cargo for retrospective studies [15]. Collectively, these features establish tumor EVs as promising complements to tissue biopsies, supporting systematic and repeatable molecular assessments for diagnosis, prognosis, and treatment monitoring.

Surface markers on tumor EVs closely mirror those of their originating tumor cells. Therefore, analyzing tumor EVs through the detection of surface markers enriched on tumor cells but absent in normal cells provides a direct and systematic approach for molecular assessment. Initial efforts were devoted to characterize single-marker EV subpopulations. For example, Melo et al. [16] demonstrated that GPC1^+^ EVs correlated with pancreatic cancer burden, while Porcelli et al. [17] reported that elevated uPAR^+^ EV levels in metastatic melanoma and were associated with resistance to immune checkpoint inhibitor therapy. Similar approaches have been applied to breast [18, 19], pancreatic [20], colorectal [21], and prostate cancers [22], confirming the clinical feasibility of EV surface marker profiling. Expanding this concept, our group has developed EV surface protein assays using click chemistry-mediated enrichment to isolate tumor EVs subpopulations defined by specific tumor surface proteins associated with hepatocellular carcinoma (HCC) [23], pancreatic ductal adenocarcinoma (PDAC) [24], ovarian cancer (OCa) [25], and other cancers [26, 27].

Recent advances have increasingly embraced *paired* and *colocalized* surface marker strategies to overcome the inherent limitations of single-marker assays, which often suffer from low specificity. By requiring the co-expression of two or more tumor-associated markers on the same EV, these approaches establish a biological “AND-gate” that significantly improves diagnostic specificity. Tian et al. [18] reported a thermophoretic aptasensor (TAS) that profiled colocalized prostate cancer EV markers, enabling sensitive detection from minimal plasma input. Similarly, Mercy BioAnalytics extended this principle to ovarian cancer [28, 29], applying proximity ligation assays (PLA) to immunomagnetically enriched EVs co-expressing MUC1 and FOLR1, functioning as a multi-marker “logic gate”. Beyond these examples, imaging-based multiplexed platforms such as Dual Imaging Single Vesicle Technology [30] have achieved single-vesicle resolution for early detection of HER2^+^ breast cancer, while nanoplasmonic profiling [31] has enabled high-throughput detection of colocalized EV markers in PDAC with improved specificity. Collectively, these advances demonstrate a paradigm shift in EV diagnostics, transforming the challenge of EV hetero-geneity into an opportunity for precise and molecularly defined liquid biopsy.

**FIGURE 1 |:**
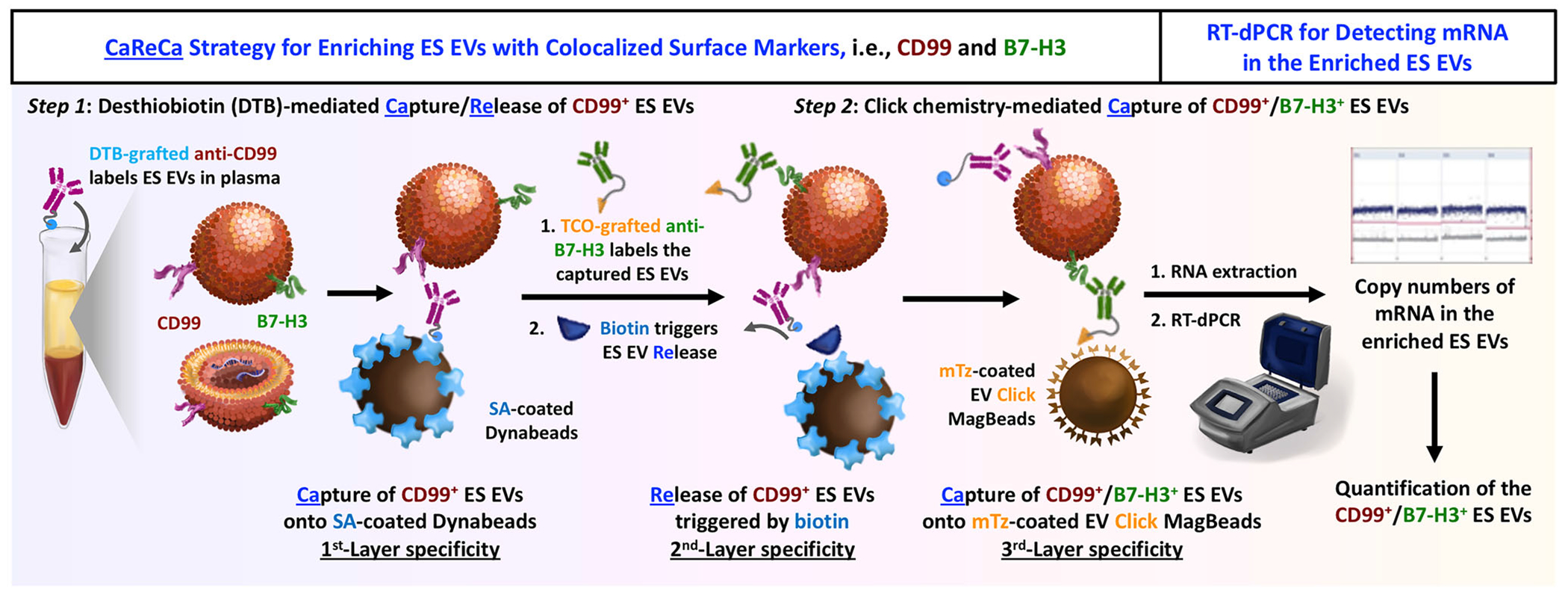
Schematic of the ES EV CaReCa Assay for Quantifying ES EVs Displaying Colocalized CD99 and B7-H3 in Patient Plasma. The two-step sequential enrichment workflow introduces three layers of molecular specificity that suppress background EV signals. ***Step 1***: DTB-grafted anti-CD99 labels CD99^+^ ES EVs in plasma, which are **Ca**ptured onto SA-coated Dynabeads (first layer of specificity). Prior to release, TCO-grafted anti-B7-H3 antibodies are added to label CD99^+^/B7-H3^+^ EVs. Exposure to biotin competitively displaces DTB, **Re**leasing the dual-labeled EVs into solution (second layer of specificity). *Step 2*: The TCO-labeled CD99^+^/B7-H3^+^ EVs undergo a bioorthogonal click reaction with mTz-coated EV Click MagBeads, selectively **Ca**pturing EVs displaying both CD99 and B7-H3 (third layer of specificity). The enriched CD99^+^/B7-H3^+^ ES EVs are then lysed, and encapsulated *ACTB* mRNA (a stable housekeeping transcript) is quantified by RT-dPCR to determine ES EV abundance. ES, Ewing Sarcoma; EV, Extracellular Vesicle; CaReCa, Capture–Release–Capture; DTB, desthiobiotin; SA, streptavidin; TCO, trans-cyclooctene; mTz, methyltetrazine; *ACTB*, *β*-actin; RT-dPCR, reverse transcription digital droplet PCR.

Herein, we introduce the Ewing Sarcoma (ES) EV Capture–Release–Capture (CaReCa) assay (Figure 1), a streamlined and cost-effective strategy for enriching and quantifying ES EVs that display colocalized CD99 and B7-H3. The workflow consists of two sequential steps: Step 1, desthiobiotin (DTB)-mediated Capture/Release of CD99^+^ ES EVs using streptavidin (SA)-coated Dynabeads (SA–Dynabeads), followed by Step 2, click chemistry-mediated Capture of CD99^+^/B7-H3^+^ ES EVs using methyltetrazine (mTz)-coated EV Click MagBeads. This two-step process introduces three layers of molecular specificity—DTB-mediated capture, biotin-mediated release, and click chemistry-mediated recapture—which collectively suppress background EV signals in plasma. The enriched CD99^+^/B7-H3^+^ EVs are then lysed, and encapsulated *ACTB* mRNA is quantified by RT-dPCR as a readout of ES EV abundance. *ACTB* mRNA [24–27], a housekeeping transcript stably expressed across diverse cell types and conditions [32], provides a robust and sensitive proxy for quantifying the limited number of CaReCa-enriched ES EVs.

These two ES EV surface markers, i.e., CD99 and B7-H3, were selected based on literature evidence, and their colocalization was validated by immunofluorescence (IF) in A673 ES cells and immunohistochemistry (IHC) on an ES tissue microarray (TMA). Scanning electron microscopy (SEM) confirmed EV immobilization on both SA–Dynabeads and EV Click MagBeads, while transmission electron microscopy (TEM) with immuno-gold labeling validated colocalization of CD99, B7-H3, and CD63, a representative EV marker. Clinically, the ES EV CaReCa assay achieved an area under the ROC curve (AUROC) of 0.98 in distinguishing ES patients (*n* = 20) from healthy donors (*n* = 20). Moreover, longitudinal monitoring of two representative ES cases demonstrated that dynamic changes in ES EV CaReCa-derived dPCR readouts closely paralleled disease progression and treatment response, consistent with PET/CT [33] and MRI [34] findings.

ES is the second most common bone malignancy in adolescents and young adults and is classified as a rare cancer by the NIH Genetic and Rare Disease (GARD) Information Center, with only ~;200 new cases diagnosed annually in the United States [35, 36]. ES tumors are characterized by fusion oncogenes, most commonly *EWS/FLI1*, which drive tumor initiation and progression. Current clinical management, consisting of neoadjuvant chemotherapy, local control (surgery and/or radiation), followed by adjuvant chemotherapy [37], has remained largely unchanged for decades. While these regimens achieve durable remissions in localized diseases, outcomes deteriorate in the presence of metastatic lesions [38]. Salvage chemotherapy carry substantial toxicity and is often initiated only after disease progression becomes apparent, highlighting a critical gap in real-time and molecularly informed disease surveillance. By selectively enriching and quantifying ES EVs co-expressing CD99 and B7-H3, the proposed ES EV CaReCa assay offers a scalable, cost-effective, and noninvasive diagnostic platform to augment current imaging modalities by offering temporal resolution, molecular specificity, and compatibility with serial sampling. As such, this technology has the potential to support earlier detection and treatment monitoring in ES patients, ultimately improving long-term outcomes for patients facing this aggressive and rare cancer.

## 2 | Results

### 2. 1 | Selection of the Paired Surface Markers—CD99 and B7-H3, Followed by Validation with ES TMA and A673 ES Cell Lines

The diagnostic performance of the ES EV CaReCa assay is fundamentally driven by the specificity, biological relevance, and co-expression profiles of its paired surface markers. Based on reported expression profiles in ES and supporting literature evidence, CD99 and B7-H3 were selected as an orthogonal marker pair for selective enrichment of ES EVs. CD99 is expressed in nearly all ES tumors [39] and serves as the gold-standard immunohistochemical marker for ES diagnosis, offering strong specificity for ES identification. In contrast, B7-H3 is expressed in >90% of pediatric sarcomas, including ES [40], but is also present on tumor-infiltrating immune cells [41], providing high sensitivity but limited tumor specificity when used alone. Mechanistically, CD99 plays a role in cell adhesion and migration, while B7-H3 functions as an immune checkpoint molecule linked to tumor immune evasion; their non-overlapping expression profiles create an orthogonal pair that minimizes false-positive detection when applied together in an AND-gate assay. Their clinical relevance is further highlighted by emerging ES immunotherapies, particularly B7-H3–targeted agents now undergoing evaluation in chimeric antigen receptor (CAR) T-cell therapy clinical trials (NCT06500819 and NCT04483778) [42].

**FIGURE 2 |:**
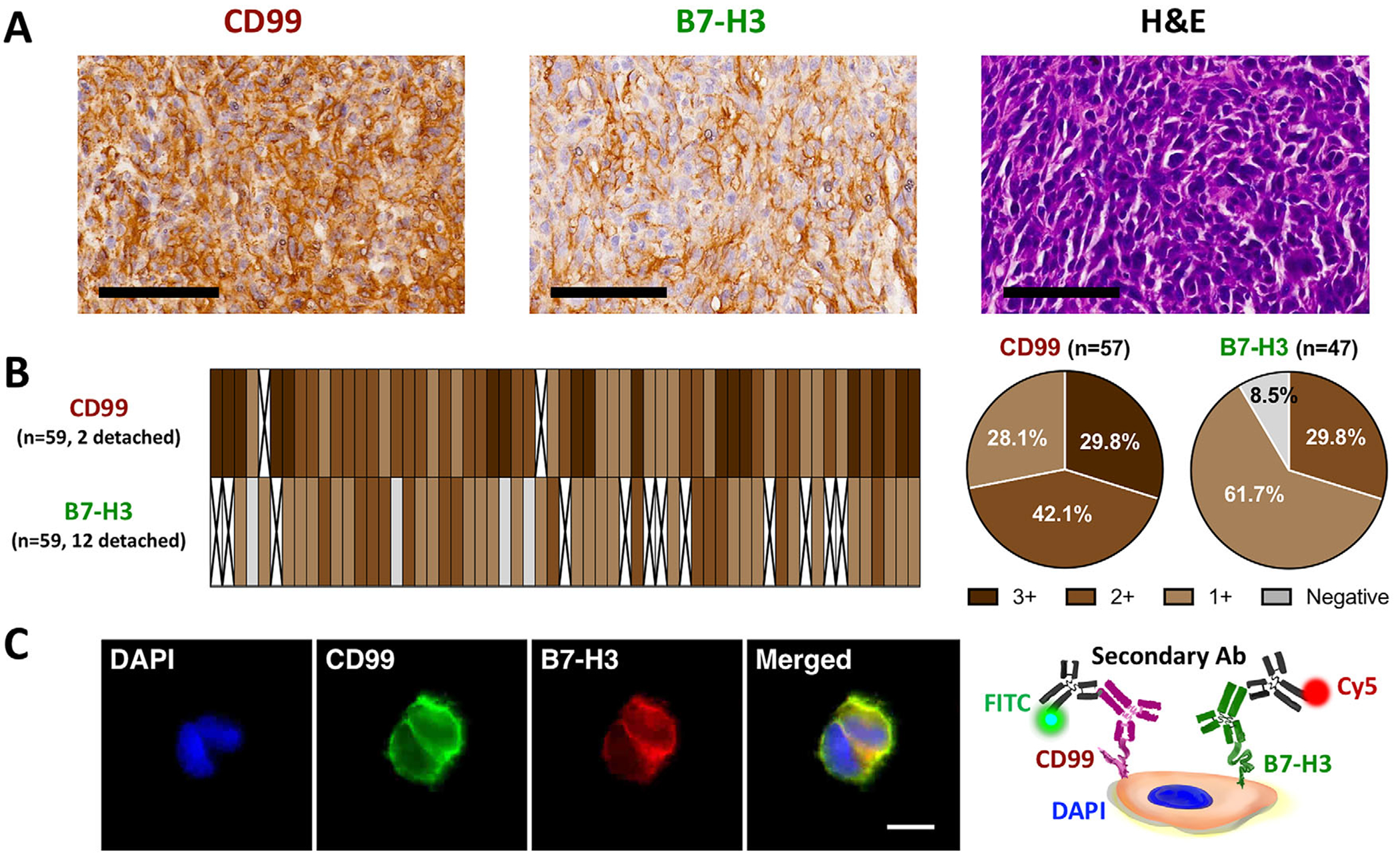
Validation of Paired Surface Markers, CD99 and B7-H3, using ES TMA and A673 ES Cells. (A) Representative H&E staining shows ES tissue morphology, while IHC staining demonstrates strong membrane expression of CD99 and B7-H3 on ES TMA slides. Scale bars = 100 μm. (B) Heatmap and pie charts (CD99, *n* = 57; B7-H3, *n* = 47) summarizing IHC staining intensities as strong (3+), moderate (2+), or weak (1+); pie charts exclude detached TMA samples. (C) Representative IF images of A673 ES cells showing colocalized membrane expression of CD99 and B7-H3, with nuclei counterstained by DAPI. Blue: DAPI; green: CD99; red: B7-H3; Schematic illustration depicts antibody binding on the ES cell surface. Scale bar = 10 μm. ES, Ewing Sarcoma; TMA, tissue microarray; H&E, hematoxylin and eosin; IHC, immunohistochemistry; ICC, immunocytochemistry; DAPI, 4′,6-diamidino-2-phenylindole.

To validate CD99 and B7-H3 expression in clinical specimens, hematoxylin and eosin (H&E) staining and IHC were performed on an ES tissue microarray (TMA) consisting of59 tumor samples (Figure 2A). Quantitative scoring is summarized in Figure 2B. For CD99, after excluding two samples that detached during processing, 17 (29.8%) showed strong staining (3+), 24 (42.1%) moderate (2+), and 16 (28.1%) weak (1+). For B7-H3, excluding 12 detached samples, 14 (29.8%), 29 (61.7%), and 4 (8.5%) of the remaining 47 evaluable samples showed moderate (2+), weak (1+), and negative staining, respectively. Overall, 91.1% of evaluable specimens were positive for both markers, demonstrating the feasibility of targeting CD99 and B7-H3 colocalization to capture ES EV subpopulations with high specificity. These findings align with previously reported expression patterns in ES [39].

We further assessed their co-expression on the cell membrane using immunofluorescence (IF) staining in A673 ES cells. The results showed strong and overlapping membrane localization of CD99 and B7-H3 (Figure 2C). Together, these findings affirm that CD99 and B7-H3 are robustly expressed in ES tissues and co-expressed at the cellular membrane, thereby validating their suitability as a paired surface marker for selective enrichment of ES EVs via the CaReCa strategy.

### 2.2. | Characterization of A673 Cell-Derived ES EVs

As a model system to validate assay performance, A673 cell-derived ES EVs were isolated from conditioned media by ultra-centrifugation. In accordance with MISEV2023 guidelines [43], the identity and structural integrity of isolated A673 ES EVs were characterized using nanoparticle tracking analysis (NTA), scanning electron microscopy (SEM), and transmission electron microscopy (TEM). NTA (Figure S1A) revealed a mean particle size of 130.32 ± 59 nm and a concentration of 8.60 × 10^10^ particles mL^−1^ in the stock EV preparation. SEM imaging (Figure S1B) showed intact, spherical morphology, and TEM imaging (Figure S1C) further confirmed the spherical or cupped vesicle structure characteristic of EVs. Together, these results verify that the isolated A673 ES EVs exhibited the expected size, morphology, and structural integrity to be exploited as a model system for the development of ES EV CaReCa assay.

### 2.3 | Linearity Study for RT-dPCR Detection of *ACTB* and EWS/FLI1 mRNA in A673 ES EVs

To determine whether *ACTB* mRNA can serve as a reliable quantitative surrogate for ES EV abundance, we performed a linearity study using serially diluted A673 ES EV samples. After lysis of EVs and RNA extraction, *ACTB* (housekeeping gene) and *EWS/FLI1* (ES-specific fusion transcript) copy numbers were quantified by RT-dPCR (Figure S1D). Both transcripts exhibited strong linear correlations with the number of EV input (R^2^ = 0.989 and R^2^ = 0.995, respectively). These findings confirm that *ACTB* mRNA quantification provides a robust and quantitative proxy for ES EV abundance, supporting its use as the molecular readout for the CaReCa assay.

### 2.4 | Characterization of DTB-grafted Anti-CD99, TCO-grafted Anti-B7-H3, and EV Click MagBeads

To evaluate the feasibility of DTB- and click chemistry-mediated ES EV enrichment, we first validated the integrity and functional reactivity of DTB-grafted anti-CD99 and TCO-grafted anti-B7-H3 antibodies using A673 cells. Cells were labeled with either DTB–anti-CD99 or TCO–anti-B7-H3, followed by staining with a FITC-labeled secondary antibody and Cy5-labeled counter motifs (SA for DTB and mTz for TCO). As shown in Figure S2A, the strong colocalization of FITC and Cy5 signals confirmed two key findings: (i) both antibodies retained specificity for CD99 and B7-H3 on the A673 cell membranes, and (ii) DTB and TCO moieties were successfully conjugated and remained intact.

In parallel, we characterized the reactivity and stability of the mTz groups on EV Click MagBeads. A click reaction with TCO-labeled Cy5 (TCO–Cy5) produced strong Cy5 fluorescence detectable by fluorescence microscopy (Figure S2B), and an image-based cytometry of the reacted beads (Figure S2C) validated the presence and reactivity of mTz groups on the surface of the EV Click MagBeads. To determine the mTz stability of the EV Click MagBeads, long-term reactivity was assessed by labeling with TCO–Cy5 over a six-month storage period. More than 60% of the initial mTz capacity was retained (Figure S2D), demonstrating the chemical robustness of mTz and supporting its suitability for reproducible EV capture.

### 2.5 | Visualization of Immobilized A673 ES EVs Following DTB- and Click Chemistry-Mediated Enrichment

**FIGURE 3 |:**
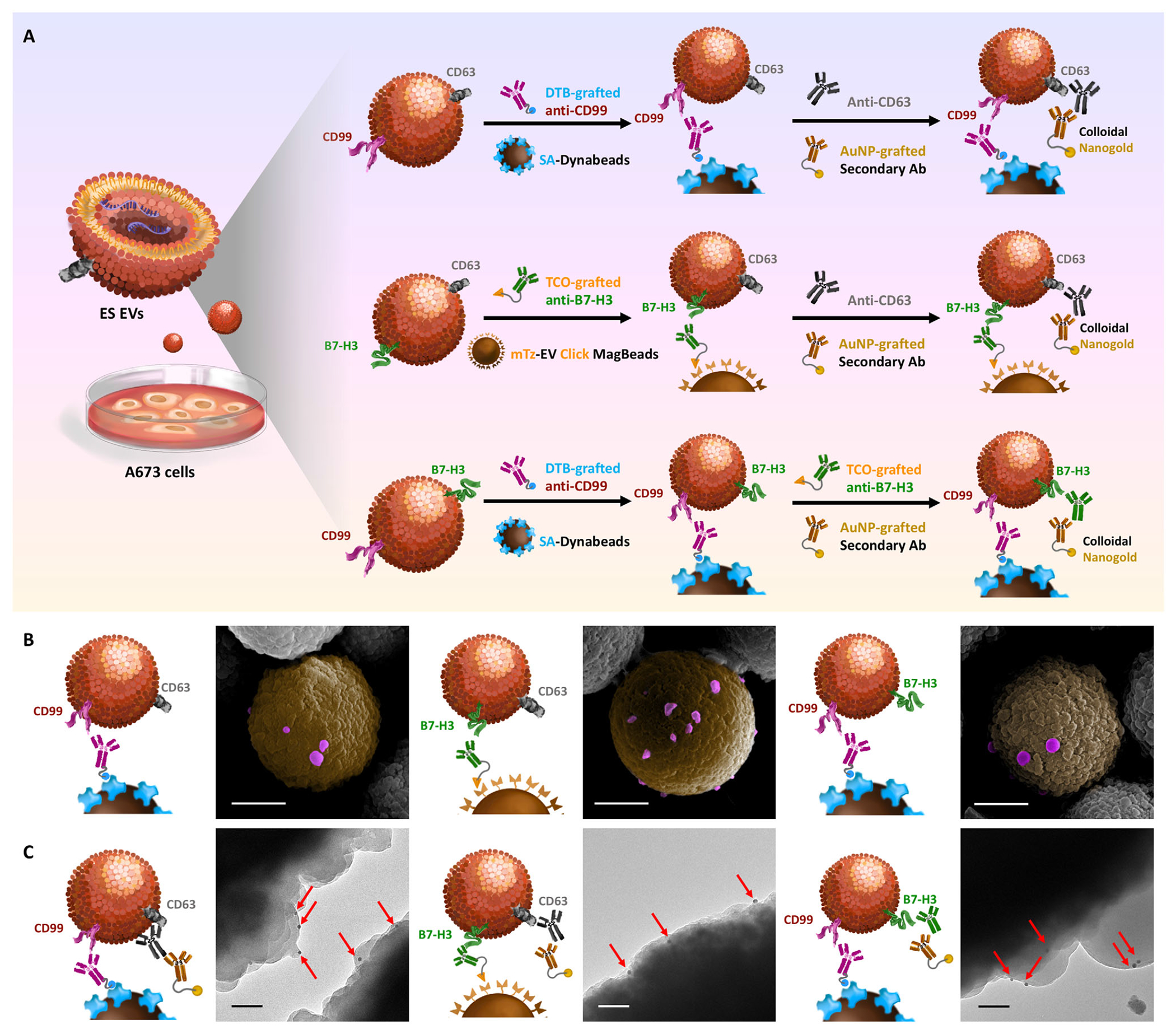
Characterization of A673-Derived ES EVs Immobilized via DTB- and Click Chemistry-Mediated Enrichment. (A) Schematic of independent enrichment of A673 ES EVs using DTB-grafted anti-CD99 with SA–Dynabeads and TCO-grafted anti-B7-H3 with EV Click MagBeads, followed by immunogold staining for CD63 or B7-H3 to confirm EV identity and marker colocalization. (B) SEM images showing immobilization of CD99^+^ and B7-H3^+^ ES EVs on SA–Dynabeads and EV Click MagBeads, respectively. Scale bars = 1 μm. (C) TEM validation of EV marker expression and colocalization: CD63 (canonical EV marker) was detected on both CD99^+^ and B7-H3^+^ EVs using 12 nm nanogold-conjugated antibodies, while B7-H3 was additionally confirmed on CD99^+^ EVs, establishing the presence of CD99^+^/B7-H3^+^ ES EVs. Scale bars = 100 nm. ES, Ewing Sarcoma; EV, Extracellular Vesicle; DTB, desthiobiotin; SA, streptavidin; TCO, trans-cyclooctene; SEM, scanning electron microscopy; TEM, transmission electron microscopy.

To independently validate the performance of the DTB- and click chemistry-mediated EV enrichment modalities, A673-derived ES EVs were captured on two orthogonal bead platforms; SA–Dynabeads using with DTB–anti-CD99 and EV Click MagBeads with TCO–anti-B7-H3 (Figure 3A). After antibody labeling, A673 ES EVs were incubated with the corresponding magnetic beads to enable selective immobilization. SEM imaging confirmed successful immobilization of CD99^+^ and B7-H3^+^ ES EVs on both platforms, with preserved spherical morphology characteristics of intact EVs (Figure 3B).

To further verify the identity of immobilized particles, bead-bound particles were immunolabeled with anti-CD63, a canonical EV surface marker, followed by nanogold-conjugated secondary antibodies (Figure 3B,C). Immunogold TEM imaging revealed distinct 12 nm gold nanoparticles on both CD99^+^ and B7-H3^+^ ES EVs immobilized on their respective bead surface, confirming CD63 expression and validating their EV origin (Figure 3C). Furthermore, to assess marker co-expression, CD99^+^ ES EVs enriched on SA–Dynabeads were probed with anti-B7-H3 and visualized by immunogold TEM. The presence of gold nanoparticles confirmed B7-H3 expression on CD99^+^ EVs, demonstrating the existence of dual-positive CD99^+^/B7-H3^+^ ES EV subpopulations (Figure 3C). These results collectively support the feasibility of using paired markers to enrich colocalized ES EVs with CD99 and B7-H3.

### 2.6 | Evaluation of DTB- and Click Chemistry-Mediated ES EV Enrichment Using Plasma Samples

We next evaluated the performance of DTB- and click chemistry-mediated enrichment of ES EVs in clinical plasma samples from ES patients (*n* = 16) and healthy donors (HD, *n* = 16) (Figure S3A). For each subject, two 100 μL plasma aliquots were processed using a modified EV surface protein assay [24]. One aliquot was enriched with DTB–anti-CD99 and SA–Dynabeads to capture CD99^+^ ES EVs, and the other was enriched with TCO–anti-B7-H3 and EV Click MagBeads to capture B7-H3^+^ ES EVs. After enrichment, EVs were lysed, and *ACTB* mRNA was quantified by RT-qPCR as a surrogate for EV abundance. Heatmap analysis (Figure S3B) revealed markedly higher *ACTB* mRNA levels in ES samples compared to HD, reflecting significantly elevated levels of both CD99^+^ and B7-H3^+^ ES EVs (Figure S3C). Receiver operating characteristic (ROC) analysis demonstrated robust diagnostic performance; CD99 achieved an AUROC of 0.84 (sensitivity 69%, specificity 94%), while B7-H3 achieved an AUROC of 0.81 (sensitivity 81%, specificity 69%) (Figure S3D). These results align with established tissue-level expression patterns (Figure 2B), CD99 offering high specificity but low sensitivity and B7-H3 offering high sensitivity with modest specificity, highlighting their complementary roles in the AND-gate architecture of the CaReCa strategy.

### 2.7 | Assessment of ES EV CaReCa Assay Using Synthetic Plasma Samples

**FIGURE 4 |:**
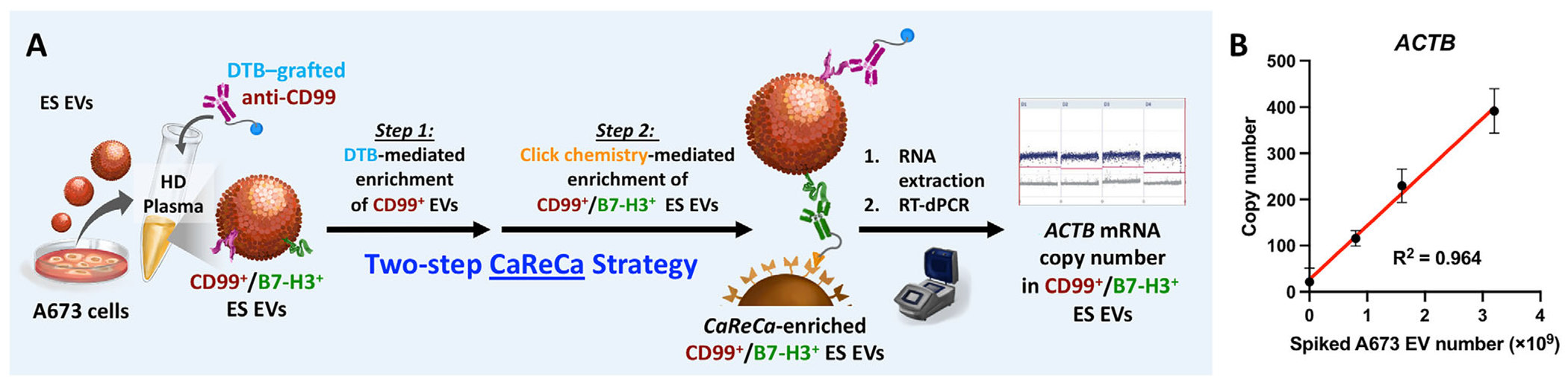
Validation of the ES EV CaReCa Assay Using Synthetic Plasma Samples. (A) Schematic workflow of the two-step ES EV CaReCa assay applied to synthetic plasma generated by spiking defined quantities of A673 ES EVs into EV-depleted HD plasma, followed by *ACTB* mRNA quantification via RT-dPCR. (B) Quantification of *ACTB* mRNA from enriched ES EVs demonstrates a strong linear correlation (R^2^ = 0.964) between *ACTB* mRNA copy number and input quantity of A673 ES EVs, confirming the assay’s quantitative performance. All data points are presented as mean ± SD (*n* = 3). ES, Ewing Sarcoma; EV, Extracellular Vesicle; CaReCa, Capture–Release–Capture; HD, Healthy Donor; *ACTB*, *β*-actin; RT-dPCR, Reverse Transcription Digital Droplet PCR; DTB, Desthiobiotin.

Having validated the DTB- and click chemistry-mediated enrichment individually, we next assessed the complete two-step ES EV CaReCa workflow using synthetic plasma samples prepared by spiking defined quantities of A673 ES EVs into EV-depleted HD plasma (Figure 4A). In ***Step 1***. DTB-mediated capture and release of CD99^+^ ES EVs, DTB–anti-CD99 was added to the synthetic plasma, and the DTB-labeled EVs were captured on SA–Dynabeads. TCO–anti-B7-H3 was then introduced to label the bead-bound CD99^+^ ES EVs before release with biotin [44, 45], providing the first two layers of assay specificity. In ***Step 2***. Click chemistry-mediated capture of CD99^+^ /B7-H3^+^ ES EVs, the released CD99^+^ ES EVs were incubated with the EV Click Mag-Beads, enabling a bioorthogonal click reaction [46, 47] between TCO-labeled EVs and mTz-functionalized beads to introduce the third layer of specificity. The enriched CD99^+^/B7-H3^+^ ES EVs were lysed, and the *ACTB* mRNA level was quantified by RT-dPCR. A strong linear correlation (R^2^ = 0.964) was observed between the *ACTB* mRNA copy number and the amount of spiked A673 ES EVs (Figure 4B), confirming the quantitative accuracy and robustness of the complete ES EV CaReCa workflow.

### 2.8 | Diagnostic Performance of ES EV CaReCa Assay in Clinical Cohorts

**FIGURE 5 |:**
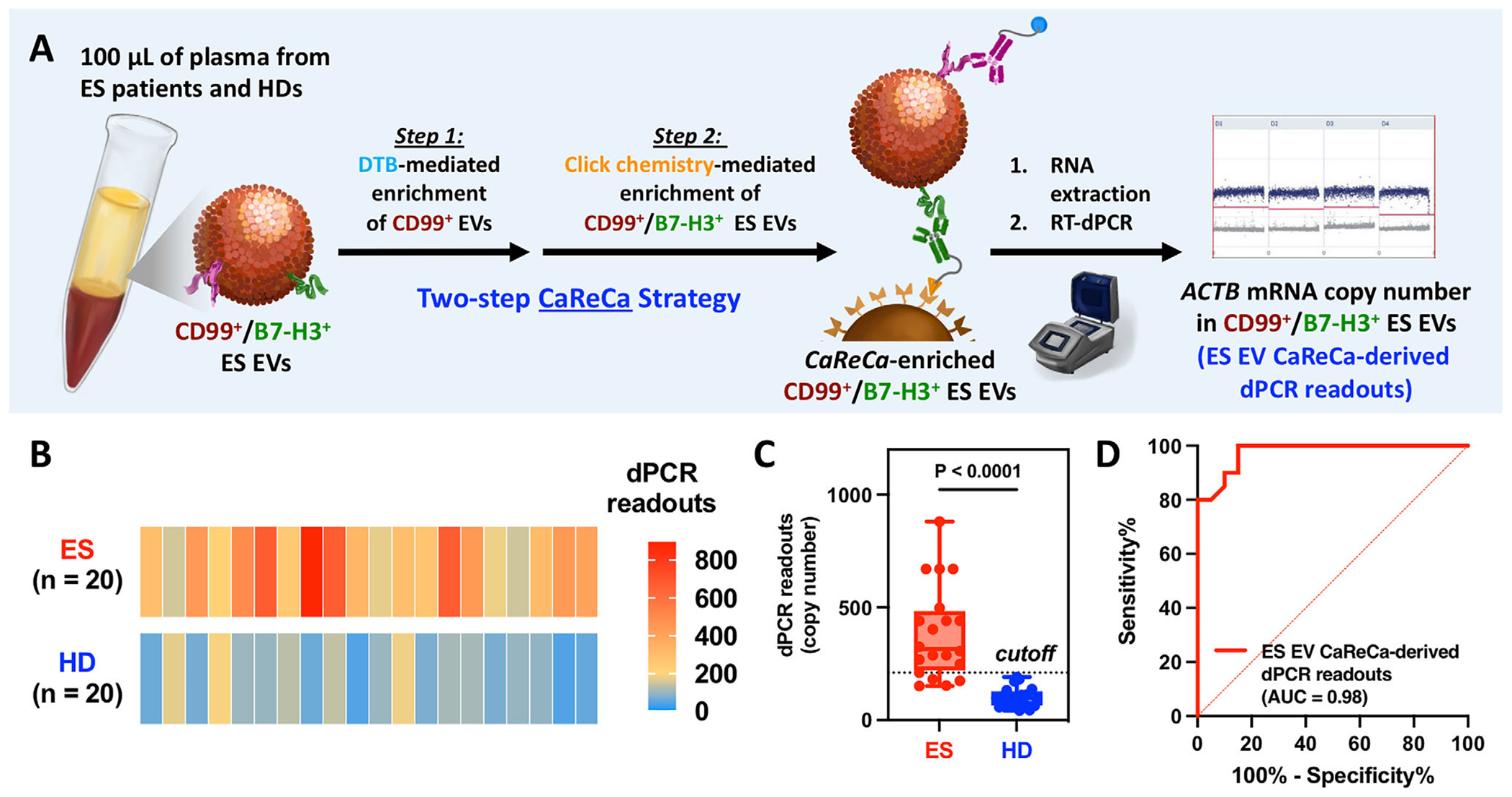
Diagnostic Performance of ES EV CaReCa Assay. (A) Schematic workflow of the two-step CaReCa assay applied to plasma (100 μL) from ES patients (*n* = 20) and HD (*n* = 20). Enriched CD99^+^/B7-H3^+^ ES EVs were lysed, and *ACTB* mRNA copy numbers were quantified by RT-dPCR. (B) Heatmap of dPCR readouts showing elevated levels of CD99^+^/B7-H3^+^ ES EVs in ES patients compared with HDs. (C) Box plot showing significantly higher dPCR readouts in ES vs. HDs (*p* < 0.0001). (D) ROC curve demonstrating high diagnostic performance (AUROC = 0.98) for distinguishing ES from HDs. An unpaired Student’s t-test was used for statistical analysis between ES and HD groups. ES, Ewing Sarcoma; EV, Extracellular Vesicle; CaReCa, Capture–Release–Capture; *ACTB*, *β*-actin; RT-dPCR, reverse transcription digital droplet PCR; HD, Healthy Donor; ROC, Receiver Operating Characteristic; AUROC, Area Under the Receiver Operating Characteristic Curve.

To determine its clinical utility, we then evaluated the diagnostic performance of the ES EV CaReCa assay in distinguishing ES patients (*n* = 20) from HDs (*n* = 20). For each subject, 100 μL of plasma was processed through the two-step CaReCa workflow (Figure 5A), and *ACTB* mRNA within the enriched CD99^+^ /B7-H3^+^ ES EVs was quantified by RT-dPCR. Clinical status was independently reviewed and confirmed by an oncologist blinded to assay results. As shown in Figure 5B,C, dPCR readouts—reflecting the abundance of CD99^+^/B7-H3^+^ ES EVs—were markedly higher in ES patients than in HDs (*p* < 0.0001; Figure 5C). ROC analysis yielded an AUROC of 0.98 (95% CI: 0.94–1.00), with 80% sensitivity (95% CI: 58%–92%) and 100% specificity (95% CI: 84%–100%) at the optimized diagnostic cutoff of 210.6 copies (Figure 5D), which was applied in all subsequent analyses. Compared with single-marker enrichment, for both CD99 (AUROC = 0.84) and B7-H3 (AUROC = 0.81) alone (Figure S3), this two-step CaReCa workflow demonstrated substantially improved discriminative power, supporting the advantage of the assay’s three-layered specificity driven by AND-gate architecture.

### 2.9 | Longitudinal Monitoring of Disease Progression and Treatment Response in ES Patients

**FIGURE 6 |:**
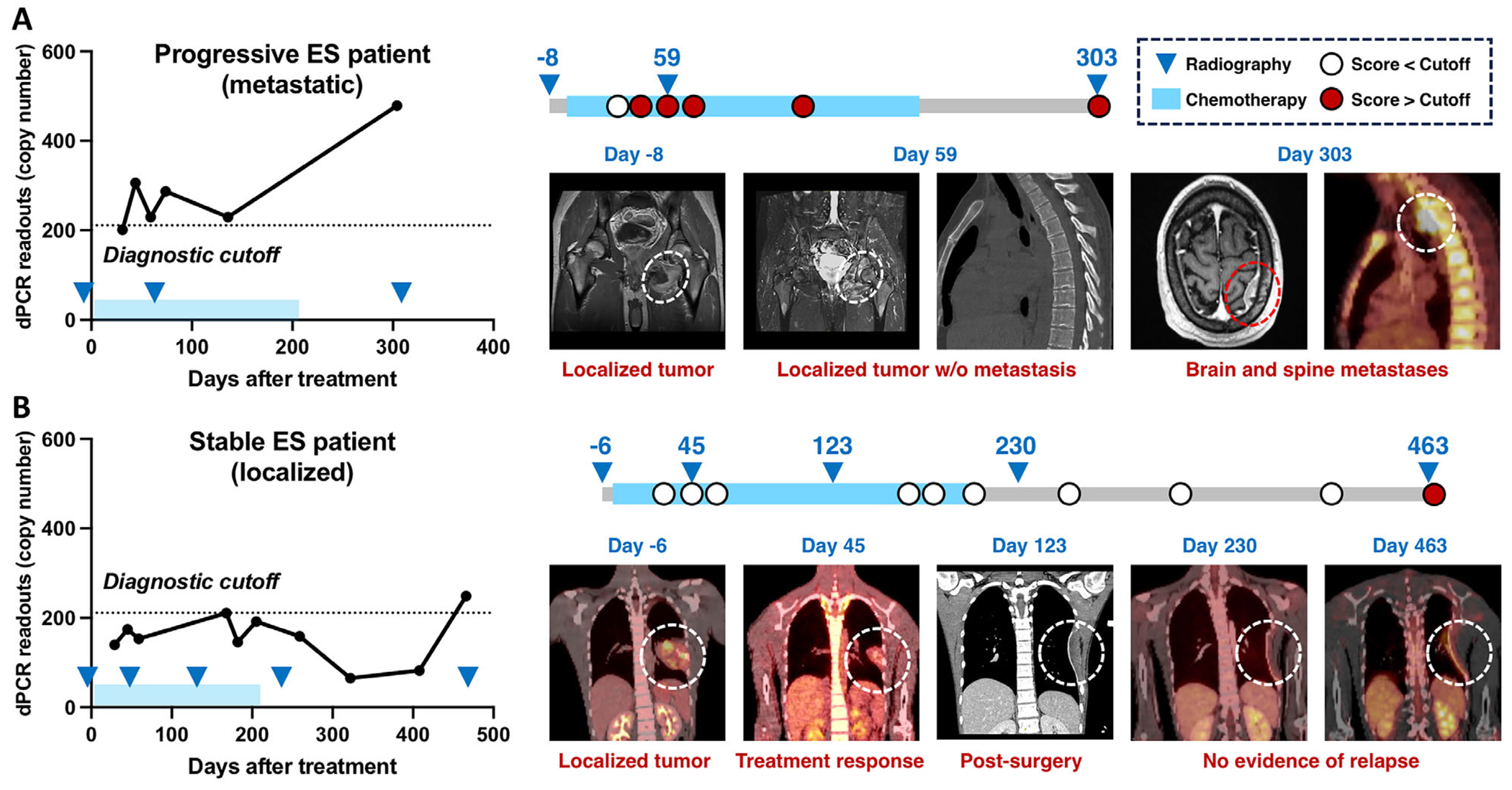
Longitudinal monitoring of ES patients using the ES EV CaReCa assay. Serial plasma samples were analyzed to quantify *ACTB* mRNA copy numbers in CaReCa-enriched CD99^+^/B7-H3^+^ ES EVs, and results were compared with PET/CT imaging during treatment and surveillance. (A) In a patient with progressive disease, dPCR readouts fluctuated over the diagnostic cutoff during chemotherapy but rose markedly after treatment completion, aligning with PET/CT evidence of metastatic spread to the brain and spine. (B) In a patient with stable disease, dPCR readouts remained below the cutoff throughout the therapy, consistent with PET/CT findings showing sustained response, successful surgery, and no relapse. ES, Ewing Sarcoma; EV, Extracellular Vesicle; CaReCa, Capture–Release–Capture; *ACTB*, *β*-actin; PET/CT, Positron Emission Tomography/Computed Tomography.

Given its robust diagnostic performance, we next evaluated whether the ES EV CaReCa assay could monitor treatment response and disease progression in real time. Serial plasma samples collected during treatment and surveillance were analyzed from two ES patients and compared with contemporaneous PET/CT findings. The same diagnostic cutoff determined by ROC analysis (210.6 copies; Figure 5D) was applied uniformly to all longitudinal samples to enable consistent interpretation of temporal changes in the dPCR readouts relative to disease burden. In the patient with progressive disease (Figure 6A), dPCR readouts fluctuated over the diagnostic cutoff during chemotherapy, indicating partial response but persistent tumor burden. After completion of chemotherapy, however, the dPCR readouts increased sharply, consistent with PET/CT evidence of metastatic progression to the brain and spine. In contrast, the patient with stable disease (Figure 6B) maintained dPCR readouts consistently below the cutoff for most of the treatment course, with a modest rise at the final time point. These results closely mirrored PET/CT imaging, which confirmed sustained treatment response, successful surgical resection, and no evidence of relapse. Together, these findings highlight the assay’s capacity to track treatment response noninvasively and detect early disease progression, offering a valuable complement to conventional imaging for longitudinal monitoring in ES patients

## 3 | Discussion and Conclusion

In this study, we established the ES EV CaReCa assay, a stream-lined and cost-effective liquid biopsy platform for enriching and quantifying ES EVs that display colocalized surface markers, i.e., CD99 and B7-H3. By detecting CD99^+^/B7-H3^+^ ES EVs, the ES EV CaReCa assay functions as a biological “AND-gate”, significantly improving diagnostic specificity compared with single-marker strategies. This two-step workflow integrates DTB-mediated capture/release with subsequent click chemistry-mediated recapture, introducing three sequential layers of molecular specificity that effectively suppress background EV signals inherent to complex biological specimens. Coupling this enrichment strategy with RT-dPCR quantification of *ACTB* mRNA, a stable and abundant housekeeping transcript, the assay circumvents the sensitivity limitations typically encountered when analyzing EVs with colocalized surface markers. Because *ACTB* mRNA levels correlate linearly with both ES EV abundance and ES-specific transcripts such as *EWS/FLI1*, it serves as a robust quantitative proxy that is resistant to sampling variability and contamination with other cell types.

In clinical validation, the CaReCa assay distinguished ES patients (*n* = 20) from HDs (*n* = 20) with an outstanding AUROC of 0.98 using only 100 μL of plasma. As expected, incorporating co-expressed markers significantly enhanced diagnostic performance relative to single-marker strategies (CD99, AUROC 0.84; B7-H3, AUROC 0.81), while preserving the complementary strengths of each marker; CD99 providing high specificity and B7-H3 offering high sensitivity. Most importantly, longitudinal analysis further demonstrated that the CaReCa assay readouts closely captured dynamic changes in disease burden throughout the treatment course, aligning with radiographic imaging. Taken together, these findings establish the ES EV CaReCa Assay as a specific, sensitive, cost-effective, and noninvasive diagnostic platform capable of augmenting conventional imaging for early detection of progression and real-time assessment of treatment response in ES patients.

Compared with existing approaches for detecting EVs with colocalized markers, such as TAS [18], PLA [28, 29], single-vesicle imaging platforms [30], and nanoplasmonic assays [31], the ES EV CaReCa assay introduces a distinctive stepwise enrichment strategy. Rather than attempting simultaneous co-detection during a single capture or imaging event, the CaReCa strategy first isolates CD99^+^ EVs, releases them, and subsequently recaptures the CD99^+^/B7-H3^+^ EV subpopulation via a rapid and bioorthogonal click chemistry. This layered “AND-gate” approach minimizes false positives from abundant irrelevant EVs while allowing high-fidelity recovery of a clinically important but low-abundance EV subpopulation from minimal sample input. Coupling this enrichment strategy with RT-dPCR quantification of encapsulated *ACTB* mRNA further enhances analytical sensitivity, addressing a key limitation of other multi-marker detection strategies that achieve high specificity at the compensated detection limits.

It is important to note that super-resolution and single-EV imaging approaches [48], including stochastic optical reconstruction microscopy (STORM) [49], total internal reflection fluorescence microscopy (TIRFM) [50], and related colocalization-based imaging techniques [51, 52], provide exceptional molecular and spatial resolution and enable direct interrogation of EV heterogeneity at the single-vesicle level. These amplification-free methods have been instrumental in advancing mechanistic understanding of EV biology and surface marker colocalization. However, such platforms typically rely on specialized instrumentation, labor-intensive sample preparation, low-throughput acquisition, and complex post-processing pipelines, which together limit their scalability and routine implementation in translational or clinical laboratory settings. Consequently, while highly valuable for discovery-driven and mechanistic studies, these approaches are less well suited for clinical applications requiring standardized workflows, reproducible quantification across large sample cohorts, or longitudinal monitoring. In contrast, the CaReCa strategy is intentionally designed to address this complementary translational need. Rather than resolving individual vesicles, the CaReCa strategy prioritizes high-specificity enrichment and sensitive population-level quantification of tumor-associated EV subpopulations through a stepwise capture–release–capture workflow. By leveraging commercially available magnetic beads, aqueous solution-phase chemistry, and RT-dPCR platforms compatible with CLIA-certified laboratories, the CaReCa strategy enables reproducible analysis from minimal sample input and is readily adaptable to automation, making it suitable for clinical deployment where throughput, robustness, and regulatory compatibility are critical.

From a biological perspective, the detection of CD99 and B7-H3 colocalized on circulating ES EVs reflects hallmark features of ES tumor biology. CD99 is expressed in nearly all ES cases [39] and remains a gold standard diagnostic marker in tissue pathology. Similarly, B7-H3 is expressed in most pediatric sarcomas including ES [42], and is under active investigation as an immunotherapy target, including in CAR T-cell trials [42]. Detecting their co-expression thus provides a clinically relevant molecular signature with diagnostic, prognostic, and therapeutic implications, particularly in the context of monitoring immunotherapy or emerging targeted therapies for ES patients.

Despite its significant promise, the ES EV CaReCa assay has several limitations. First, the relatively small cohort sizes limit the strength of conclusions pertaining to treatment monitoring, and the longitudinal analyses presented in this study (*n* = 2) are intended to illustrate assay feasibility and potential clinical utility rather than to establish statistically definitive prognostic performance. As ES is classified as a rare cancer [35, 36], this study nonetheless represents an important step toward clinical validation. Continued collection of clinically well-annotated patient samples, along with future multi-institutional studies involving larger cohorts, will enable more rigorous assessment of predictive performance and refinement of actionable thresholds. Additionally, as both CD99 and B7-H3 are being explored as therapeutic targets, the CaReCa assay may serve as a companion diagnostic or pharmacodynamic monitoring tool in immunotherapy trials, enabling longitudinal noninvasive assessment of target expression dynamics over time. Ongoing work is focused on further optimizing the CaReCa strategy through explicit analytical validation, including determination of limit of detection (LoD) and limit of blank (LoB) using positive and negative controls, as well as systematic evaluation of sensitivity, specificity, reproducibility, and stepwise capture/release efficiency, which will enable clinical translation of the assay. In parallel, we are extending the CaReCa framework beyond Ewing sarcoma by identifying appropriate paired surface markers for additional malignancies, which will help define the broader applicability of the CaReCa strategy across malignancies with greater confidence.

In conclusion, the ES EV CaReCa assay offers a sensitive, specific, noninvasive, and clinically translatable liquid biopsy tool for ES. Its capability to deliver high diagnostic accuracy from small plasma volumes, combined with its potential for real-time disease monitoring, highlights the assay’s clinical utility as a complementary approach to conventional radiographic imaging. Future optimization and large-scale clinical validation will be essential to advance this assay toward integration into routine clinical practice for ES and potentially other malignancies.

## 4 | Experimental Section

### 4.1 | Instrumentation

Nanoparticle tracking analysis (NTA) was performed using a ZetaView PMX-x35 (Particle Metrix, Germany). Fluorescence readouts were measured using a CLARIOstar microplate reader (BMG Labtech, Germany).

### 4.2 | Scanning Electron Microscopy (SEM) Imaging

SEM images were obtained using a Teneo SEM (FEI, USA) at 10 kV. ES EVs and EV-immobilized magnetic beads were placed onto silicon wafers and air-dried overnight at room temperature. Samples were sputter-coated (Pelco SC-7, Pelco, USA) with a gold target (Ted Pella, USA) before imaging.

### 4.3 | Transmission Electron Microscopy (TEM) Imaging

TEM images were obtained using a Tecnai 12 Quick Cryo-EM (FEI, USA). A carbon-coated copper grid (400 mesh; Ted Pella, USA) was glow discharged with the PELCO easiGlow system (Ted Pella, USA) to enhance hydrophilicity and used within 1 h. ES EV or EV-immobilized magnetic beads were placed onto the treated grid and incubated for 2 min at room temperature. Excess samples were removed by blotting with filter paper, followed by three washes with filtered 5% uranyl acetate. Samples were stained with filtered 5% uranyl acetate for 1 min. For unstained samples, distilled water was substituted for the uranyl acetate solution.

### 4.4 | Clinical Blood Sample Processing

Peripheral venous blood samples from ES patients (Table S1) were collected through the UCLA Pediatric Bone and Soft Tissue Sarcoma Program. Blood samples from healthy donors (Table S2) were collected from UCLA. All samples were collected with written informed consent under IRB-approved protocols (IRB #19-000857). Blood samples were drawn into BD Vacutainer EDTA tubes (cat. #366643) and processed within 4 h. Briefly, plasma was isolated by centrifugation at 500 *g* for 10 min, followed by 4,600 *g* for 10 min to remove cells and debris. Plasma aliquots were stored at −80°C to minimize freeze-and-thaw cycles.

### 4.5 | ES Cell Line Culture

The A673 cell line (RRID: CVCL_0080) was purchased from American Type Culture Collection (ATCC). Mycoplasma contamination was routinely tested using the MycoAlert kit (Lonza). Cells were cultured in Dulbecco’s Modified Eagle’s Medium (DMEM; ATCC) supplemented with 10% fetal bovine serum (FBS) and 100 U mL^−1^ penicillin-streptomycin (Thermo Fisher) in a 5% CO_2_ incubator.

### 4.6 | Immunofluorescence (IF) Staining Image

A673 cells cultured on glass coverslips were fixed with 4% paraformaldehyde (PFA) for 30 min, permeabilized with 0.05% Triton X-100 for 10 min, and blocked with 2% donkey serum (Jackson ImmunoResearch) for 30 min. Cells were then incubated overnight at 4°C with anti-human CD99 or anti-human B7-H3 primary antibodies prepared in PBS containing 2% donkey serum. After washing with PBS, cells were stained with DAPI (1:1000 v/v; Invitrogen) and secondary antibodies (FITC anti-goat IgG and Cy5 anti-rabbit IgG; 1:500 v/v; Invitrogen). Images were obtained using a Nikon Eclipse 90i fluorescence microscope with 40× objective.

### 4.7 | Tissue Microarray (TMA) for Immunohistochemistry (IHC)

Formalin-fixed, paraffin-embedded (FFPE) tumor tissue from 59 ES patients diagnosed between 2015–2025 were collected at Zhongshan Hospital, Fudan University. TMA blocks were prepared and serial 4 µm sections were mounted on poly-l-lysine–coated slides. Hematoxylin and eosin (H&E) staining and IHC were performed on a Ventana Benchmark ULTRA automated system using validated and optimized protocols for anti-human CD99 and anti-human B7-H3 antibodies (R&D Systems). Two independent pathologists scored staining intensity as strong (3+), moderate (2+), weak (1+), or negative (0) by. TMA cores with tissue detachment or noncancerous morphology were excluded from analysis.

### 4.8 | Collection of A673 ES EVs from Cell Culture Medium

A673 cells were cultured in 18 Nunc EasYFlask Flasks (175 cm^2^, Thermo Fisher) to 80% confluency. Before EV collection, culture medium was replaced to serum-free medium (13 mL per flask) and cells were incubated for 24 h under starvation. Conditioned medium was collected and centrifuged at 300 *g* for 10 min at 4°C, followed by 2800 *g* for 20 min at 4°C to remove cell debris. Supernatants were gently transferred to Ultra-Clear Tubes (38 mL per tube; Beckman Coulter, USA) and ultracentrifuged at 100 000 *g* for 90 min at 4°C. EV pellets were resuspended in 400 μL PBS and regarded as EV stock.

### 4.9 | Characterization of ES EVs

ES EVs size distribution and concentration were measured using NTA. Samples were diluted 100–10,000-fold in 0.22 μm-filtered PBS, and each sample was measured in triplicate. For electron microscopy (EM) imaging, EVs were fixed in 4% PFA for 30 min and prepared for SEM/TEM as described above.

### 4.10 | Synthesis of Methyltetrazine/PEG-Grafted Dynabeads (EV Click MagBeads)

EV Click MagBeads were prepared following our recent reports [53, 54]. Dynabeads M-270 amine (6 mg, 200 μL) were washed twice with DMSO and resuspended in 200 μL DMSO containing triethylamine (90 μmol). The beads were reacted with mTz-PEG4-NHS (0.9 μmol) for 60 min, followed by mPEG4-NHS (18 μmol) for another 60 min. Residual NHS esters were quenched with 120 μL Tris buffer (pH 8.4) for 10 min. The beads were then washed three times with distilled water, dried under vacuum at room temperature, and stored at 4°C with protection from light. Before use, dry beads were reconstituted in TET buffer (TE + 0.05% Tween-20).

### 4.11 | Characterization of EV Click MagBeads

The mTz content on EV Click MagBeads was quantified via back-titration using TCO–Cy5. A standard fluorescence curve for TCO–Cy5 (0–5 μm) was generated (Ex/Em = 620 nm/680 nm). EV Click MagBeads (0.5 mg) were incubated in 200 μL of 5 μm TCO–Cy5 for 1 h at room temperature. After magnetic separation, the fluorescence decrease in the supernatant was used to determine the amount of TCO–Cy5 bound to the beads.

### 4.12 | Synthesis of DTB-Grafted Anti-CD99 (DTB–Anti-CD99)

Anti-human CD99 (BSA- and azide-free; 1 mg mL^−1^, 20 μL) was incubated overnight at 4°C with sulfo-NHS-LC-desthiobiotin (1 mm, 2.7 μL; Thermo Fisher) in 100 μL PBS (pH 7.4) with gentle shaking. Excess NHS ester was removed using a Zeba 40 kDa desalting column. Antibody concentration was determined via absorbance at 280 nm (A_280_) using NanoDrop One.

### 4.13 | Synthesis of TCO-Grafted Anti-B7-H3 (TCO–Anti-B7-H3)

Anti-human B7-H3 (BSA- and azide-free; 1 mg mL^−1^, 20 μL) was incubated overnight at 4°C with TCO-PEG4-NHS (1 mm, 2.7 μL; VectorLab) in 100 μL PBS (pH 7.4) with gentle shaking. Excess NHS ester was removed using a Zeba 40 kDa desalting column. The final antibody concentration was determined by A_280_. The degree of labeling (DOL) of TCO was quantified by reacting with Cy5-labeled methyltetrazine (Cy5–mTz) and analyzing Cy5 absorbance on a NanoDrop, yielding ~3 TCO per antibody across the entire batch.

### 4.14 | Immunogold TEM Imaging of ES EV-Immobilized Magnetic Beads

A673 ES EV-immobilized SA–Dynabeads (via DTB–anti-CD99) and EV Click MagBeads (via TCO–anti-B7-H3) were prepared using synthetic plasma as described in the CaReCa-mediated enrichment of ES EVs section. The beads were washed with PBS and fixed in 4% PFA for 30 min. For the identification of CD63 (EV marker), the fixed beads were incubated with anti-human-CD63 (mouse, 1:50 v/v; Abcam) in 1% BSA for 1 h. For the identification of CD99^+^/B7-H3^+^ ES EVs, the CD99^+^ EV-bound SA–Dynabeads were incubated with anti-B7-H3 (goat, 1:50 v/v) in 1% BSA for 1 h. At room temperature, after washing twice with PBS, the bead samples were incubated with 12 nm nanogold-conjugated secondary antibodies (anti-mouse for CD63 or anti-goat for B7-H3, 1:20 v/v; Jackson ImmunoResearch) in 1% BSA for 30 min and washed twice with PBS. The nanogold-labeled samples were placed onto glow discharged TEM grids and prepared for imaging without staining, following the procedure detailed in the *TEM imaging* section.

### 4.15 | Preparation of Synthetic Plasma Samples

Synthetic plasma samples mimicking the plasma of ES patients were prepared to validate the assay’s reliability for detecting ES EVs in plasma conditions. EV-depleted HD plasma was prepared by ultracentrifugation. In brief, 38 mL HD plasma per tube (six tubes total) was ultracentrifuged at 100 000 *g* for 90 min at 4°C. The top two-thirds (~26 mL) were carefully collected as EV-depleted HD plasma. The synthetic plasma samples were prepared by spiking the desired amount of A673 EVs into EV-depleted HD plasma.

### 4.16 | CaReCa-mediated Enrichment of ES EVs from Plasma Samples

ES EVs were enriched from 0.1 mL synthetic or clinical plasma samples via a two-step capture workflow. First, 100 ng DTB–anti-CD99 (in 100 μL TE buffer) was added to 100 μL plasma sample and incubated for 45 min at room temperature on an orbital shaker. DTB-labeled EVs were captured by adding 100 μg SA–Dynabeads (pre-blocked with 10% BSA in TE buffer) and incubating for 45 min with gentle agitation. Beads were magnetically isolated, washed with 500 μL TET buffer, then bead-bound EVs were eluted into 50 μL of TE buffer containing 0.5 mM biotin and while simultaneously labeling with 25 ng TCO–anti-B7-H3 for 1 h at 37°C with shaking. After magnetic removal of SA–Dynabeads, the supernatant containing DTB- and TCO-grafted ES EVs was transferred into 50 μg EV Click MagBeads (pre-blocked with 10% BSA) and incubated for 45 min with agitation. Finally, EV Click MagBeads were recovered and washed three times with 500 μL TET buffer to yield purified and immobilized CD99^+^/B7-H3^+^ ES EVs on the beads.

### 4.17 | Quantification of *ACTB* mRNA by RT-dPCR

The purified ES EVs were lysed in 700 μL QIAzol (Qiagen, USA). Total RNA was isolated using the miRNeasy Micro Kit (Qiagen, USA) and cDNA was generated with the Maxima H Minus First Strand cDNA Synthesis Kit (Thermo Scientific, USA). The resulting cDNA was quantified a QX200 Droplet Digital PCR System (Bio-Rad, USA) using TaqMan probes (Thermo Scientific, USA). Absolute copy numbers of the transcript were calculated using QuantaSoft software.

### 4.18 | Statistical Analysis

Data were reported as mean ± SD. Statistical differences among groups were calculated by one-way ANOVA with Tukey’s multiple comparison tests or Student’s unpaired t-test, depending on the number of groups. The optimal cutoff for ROC analyses was determined for maximizing sensitivity and specificity.

## Supplementary Material

supporting info

Additional supporting information can be found online in the Supporting Information section.

**Supporting File:** adhm70974-sup-0001-SuppMat.pdf.

## Data Availability

The data that support the findings of this study are available from the corresponding author upon reasonable request.
