# Supplementary material for: Detection of Extracellular Vesicles with Colocalized Surface Markers via a Capture–Release–Capture Strategy for Treatment Monitoring in Ewing Sarcoma
Source: Adv Healthc Mater. Author manuscript; Available in PMC 2026 Apr 15. (PMC13078352; doi:10.1002/adhm.202505917)
Supplement: supporting info [file NIHMS2158055-supplement-supporting_info.pdf]

Supporting Information

Detection of Extracellular Vesicles with Colocalized Surface Markers via a Capture–Release–Capture Strategy for Treatment Monitoring in Ewing Sarcoma

You-Ren Ji+, Yong Ju+, Hui Kong, Yaya Xu, Chen Zhao, Ryan Zhang, Yue Ma, Lynn L. Zheng, Lucy R. Shi, Alex Wu, Lu-An Lin, Carina Peijia Wu, Audrey Qian, Emily Ren, Christine Zhang, Kenny Vo, Sarah Dry, Joseph G. Crompton, Noah Federman, Yazhen Zhu, Steven J. Jonas*, Hsian-Rong Tseng*, Shaohua Lu*, Junseok Lee*

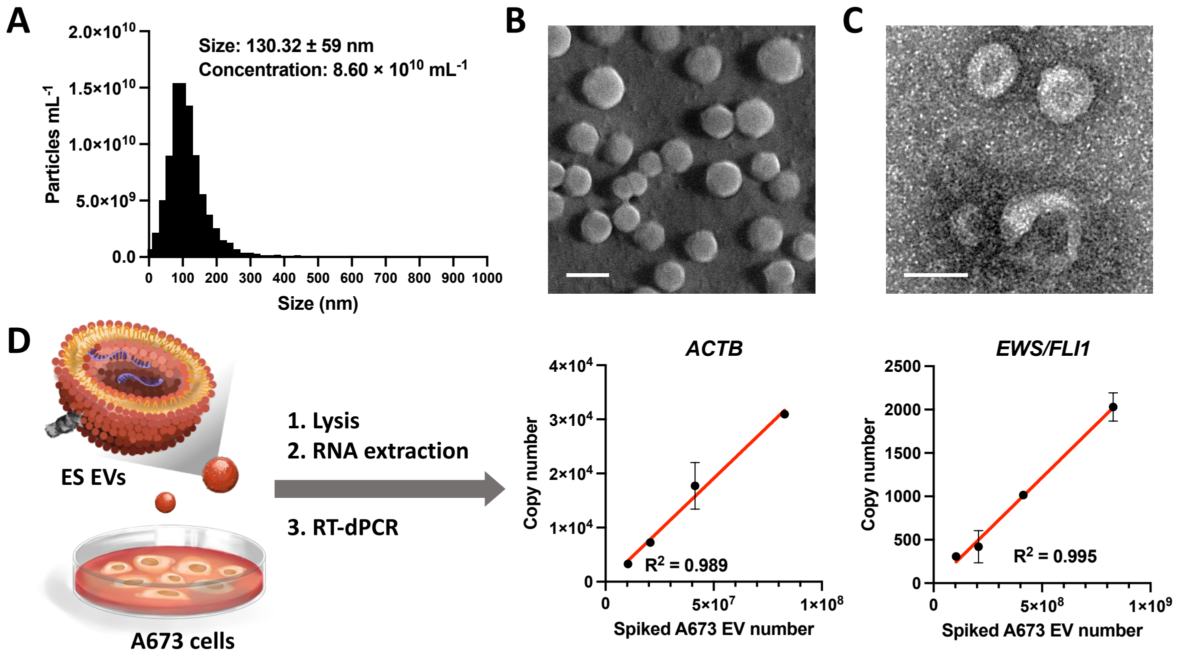

Figure S1. Characterization of A673 ES EVs. (A) Size distribution and concentration of A673 ES EVs measured by NTA. (B–C) Morphology of ES EVs observed by (B) SEM and (C) TEM imaging. Scale bars = 100 nm. (D) Linearity study for RT-dPCR detection of *ACTB* and *EWS/FLI1* mRNA in A673 ES EVs. All data points are presented as mean ± SD (n = 3).

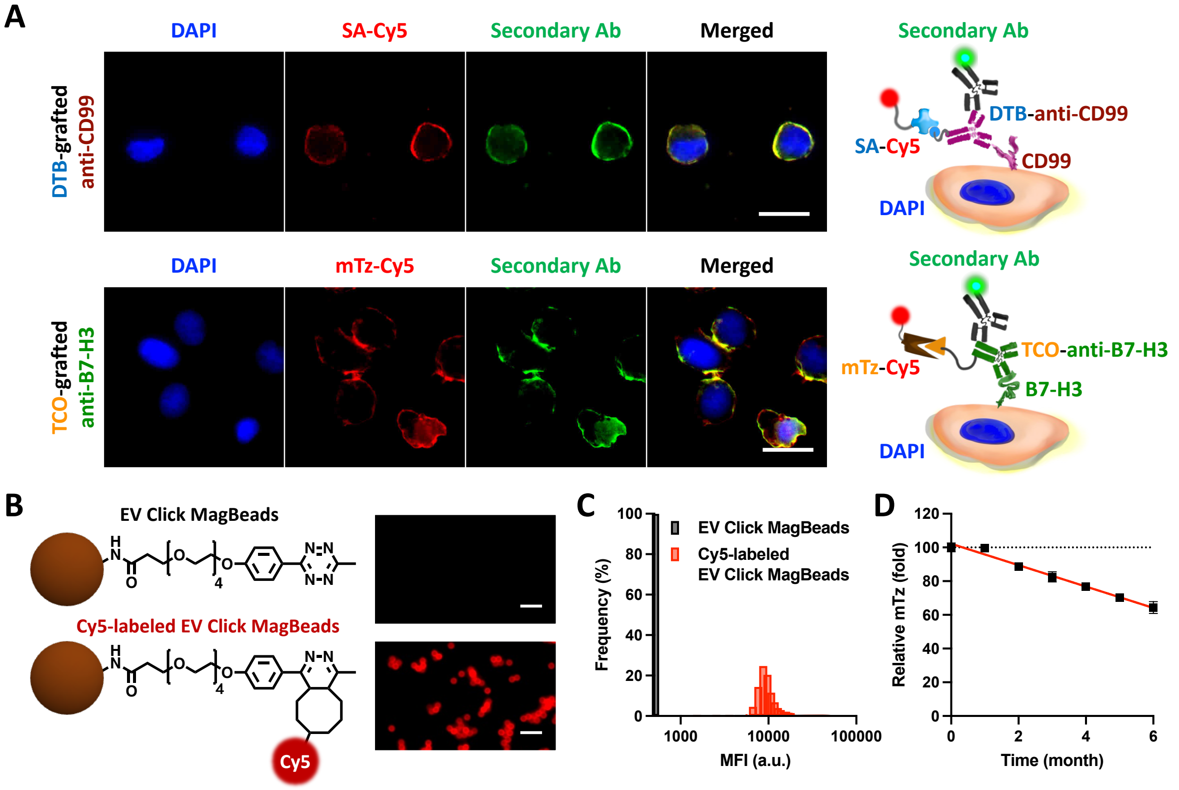

Figure S2. Characterization of antibodies and EV Click MagBeads. (A) Validation of integrity of DTB–anti-CD99 and TCO–anti-B7-H3 antibodies by immunofluorescence staining of A673 cells. Scale bars = 10 µm. (B) Fluorescence micrographs of EV Click MagBeads before and after labeling with TCO-Cy5 (scale bars = 10 µm) and (C) corresponding histograms of mean fluorescence intensity (MFI). (D) Lifetime of the mTz group on EV Click MagBeads. All data points are presented as mean ± SD (n = 3).

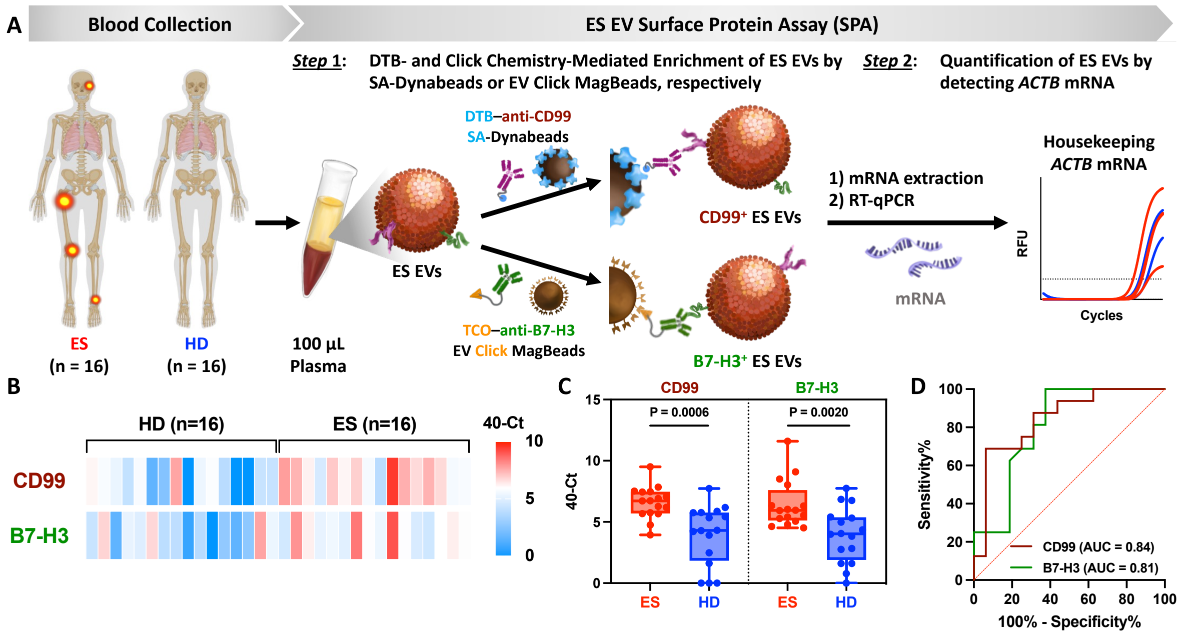

Figure S3. Evaluation of DTB- and click chemistry-mediated ES EV enrichment using plasma samples. (A) A general workflow of ES EV surface protein assay for evaluating DTB-mediated CD99^{+} ES EV enrichment and click chemistry-mediated B7-H3^{+} ES EV enrichment. After enrichment of each EV subpopulation, EV-encapsulated *ACTB* mRNA was quantified by RT-qPCR. (B) Heatmap, (C) box plot (mean ± SD), and (C) ROC curve of each ES EV subpopulation to distinguish ES patients (n = 16) from healthy donors (HD; n = 16). Unpaired Student's t-test was used for statistical analysis between ES and HD groups.

Table S1. Clinical characteristics of the ES patients for the CaReCa assay.

**Characteristic**	*n = 20*
**Age, median (IQR)**	13 (12–19)
**Gender**	
Male, *n*	12 (60%)
Female, *n*	8 (40%)
**Disease status at diagnosis**	
Localized	15 (75%)
Metastatic	5 (25%)

Table S2. Clinical characteristics of HDs for the CaReCa assay.

**Characteristic**	*n = 20*
**Age, median (IQR)**	23 (21–25)
**Gender**	
**Male, *n* (%)**	9 (45%)
**Female, *n* (%)**	11 (55%)
